# Surgical and Prosthetic Management of a Peripheral Giant Cell Granuloma Around Dental Implants: A Case Report and Review of the Literature

**DOI:** 10.7759/cureus.96837

**Published:** 2025-11-14

**Authors:** Ioannis Michalis, Konstantinos Tasiopoulos, Nefeli Christou, Ioanna Mitsika, Spyridon Silvestros

**Affiliations:** 1 Department of Dentistry, National and Kapodistrian University of Athens, Athens, GRC

**Keywords:** bone loss, dental implants, peri-implant benign fibrous tumor, peri-implant lesions, peripheral giant cell granuloma

## Abstract

A peripheral giant cell granuloma (PGCG) is a benign fibrous connective tissue entity described as a tumor-like hyperplasia that appears on the gingiva and alveolar ridges. Histopathologically, a PGCG is characterized by the presence of multinuclear giant cells and originates from periodontal membrane or periosteal cells. PGCG is considered a reactive lesion resulting from chronic traumatic or irritating factors, such as calculus accumulation, food impaction, or inadequate dental restorations. The purpose of this article is the presentation of a case of PGCG in an edentulous area around dental implants in the mandible of a male patient with no recurrence in a two-year follow-up.

A 59-year-old, non-smoking male patient presented to the clinic for evaluation of a tumor-like lesion in the left side of the mandible that had appeared 1.5 months prior to the visit. The medical history consisted of pre-diabetes and hypertension, while his dental history included chronic periodontitis, which had led to the loss of his natural teeth in the infected area and their replacement with implant-supported restoration. Clinical evaluation revealed an asymptomatic, soft-consistency, red/purple tumor-like mass with a broad base, measuring 1.5-2 cm in diameter. The lesion was located on both the buccal and lingual aspects of the alveolar ridge in the mandibular region in positions #34-#35. Radiographic evaluation with periapical radiographs revealed no significant findings.

Although implant-related PGCG remains a rare complication in the context of the widespread use of dental implants, its incidence may be underestimated due to underreporting and the common omission of histopathological analysis following excision of peri-implant soft tissue lesions. A possible contributing factor is that peri-implant soft tissue lesions are sometimes excised without being referred for histopathological examination. Since PGCG can be difficult to differentiate from other reactive lesions, such as pyogenic granuloma, histopathological evaluation remains essential. Further studies with larger sample sizes are needed to better understand the etiopathogenesis and optimal treatment strategies for implant-associated PGCG.

These findings highlight the importance of increased awareness among clinicians, both in recognizing suspicious peri-implant lesions and in adopting surgical protocols that include complete excision with curettage and histological examination.

## Introduction

Peripheral giant cell granuloma (PGCG) is a benign fibrous connective tissue entity described as a tumor-like hyperplasia that exclusively appears on the gingiva and alveolar ridges. Clinically, the lesion may present as soft or elastic in consistency, varying in size, and either sessile or pedunculated. Its color ranges from red to bluish-purple, depending on the quantity and distribution of hemosiderin granules within the tissue.

Histopathologically, PGCG is characterized by the presence of multinuclear giant cells and originates from periodontal membrane or periosteal cells. It is not considered a particularly common lesion. Epidemiological data show that it can appear at all ages, with the peak of its appearance ranging between 30 and 40 years of age. Additionally, there is a slightly higher incidence in females, with approximately 60% of cases occurring in women [1]. According to Chaparro-Avendaño et al. (2005), the male-to-female ratio in the occurrence of PGCG is approximately 1:1.5 [1]. The cause of this female predominance remains unclear; however, sex hormones such as estrogen and progesterone are thought to play a positive role in the frequency of occurrence of similar reactive lesions [2]. There are no epidemiological population-based data available to draw definitive conclusions regarding the incidence of this entity. Nevertheless, findings from large case series provide valuable insights. In the study by Buchner et al. (2010), out of a total of 25,106 biopsies, 1,675 reactive lesions were identified, with PGCG representing the minority, 314 cases (18.7%), which accounted for only 1.25% of all biopsies examined [3]. Similarly, other studies have reported varying frequencies: Shirani et al. (2008) observed a prevalence of 6.8% in a cohort of 8,382 biopsies [2], while Motamedi et al. (2007) reported the same frequency (6.8%) in their series. In contrast, Giansanti et al. (1969) documented a considerably lower prevalence of 0.5% [4,5].

PGCG is considered a reactive lesion resulting from chronic traumatic or irritating factors, such as calculus accumulation, food impaction, or inadequate dental restorations [6]. While it commonly develops in areas adjacent to teeth or edentulous ridges, its occurrence around dental implants has also been documented. Specifically, 37 cases of implant-associated PGCG have been reported in the literature [7], and although this number remains limited, such lesions demonstrate a relatively high recurrence rate. According to the recent histopathological study by Morais et al. (2019) [8], certain differences were observed in microvessel density, proliferative activity, and the expression of CD68 and Bcl-2 among conventional PGCG and peri-implant PGCG. The presentation of a single case of PGCG can be particularly valuable for the literature, as it contributes to the refinement of diagnostic accuracy. Detailed documentation of the clinical, radiographic, and histopathological features allows clinicians to better recognize similar presentations in future patients, reducing the risk of misdiagnosis.

The purpose of this article is the presentation of an interesting case of PGCG in an edentulous area around dental implants in the mandible of a male patient with no recurrence in a two-year follow-up.

This case was previously presented as a poster at EuroPerio11, held in Vienna, Austria, from May 14 to May 17, 2025.

## Case presentation

**Figure 1:**
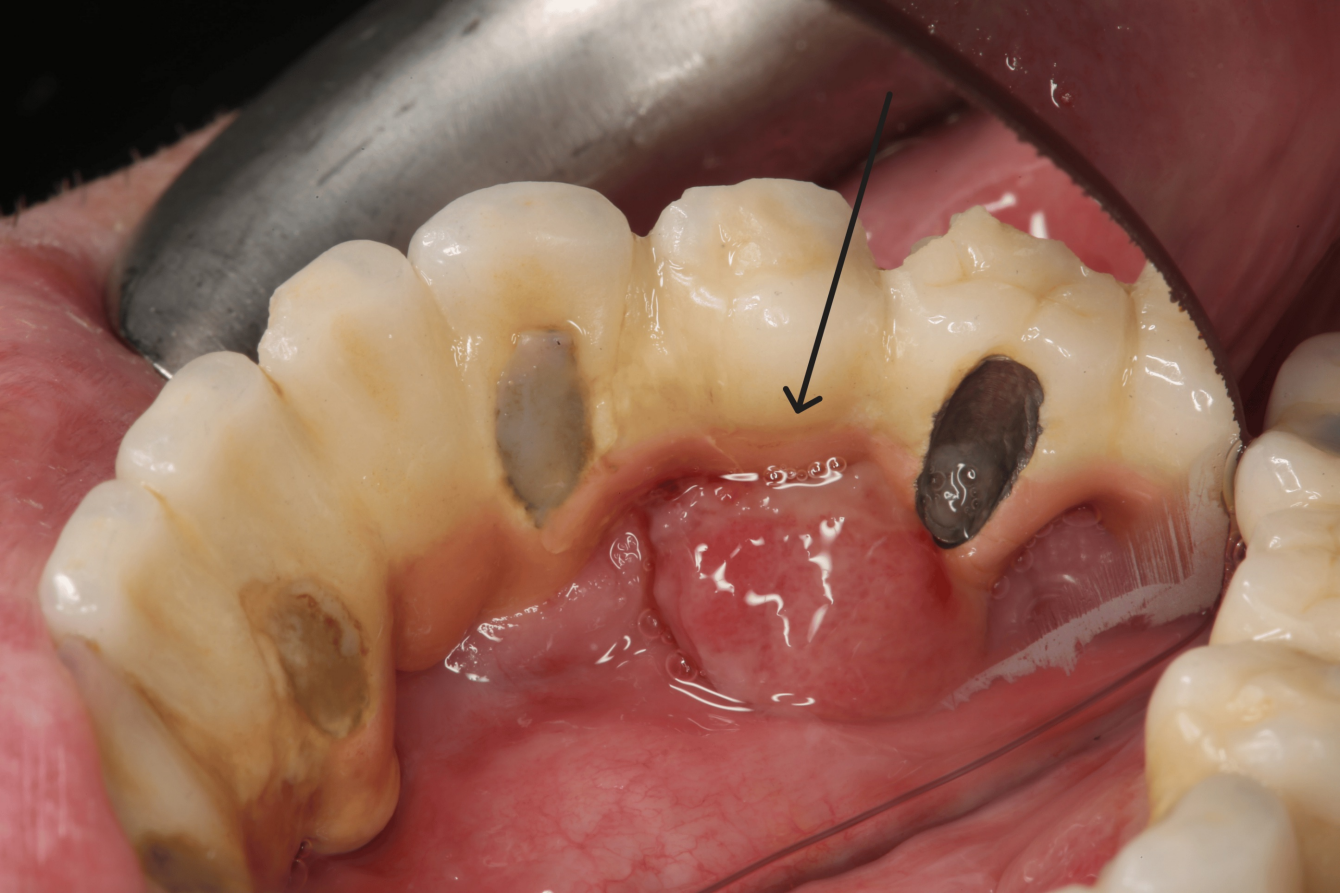
Clinical image of the lesion before the removal of the prosthetic restoration.

A 59-year-old, non-smoking male patient presented to the clinic for evaluation of a tumor-like lesion in the left side of the mandible that had appeared 1.5 months prior to the visit. The patient’s medical history included pre-diabetes and hypertension, while his dental history was notable for chronic periodontitis, which had led to the loss of his natural teeth in the affected area and their replacement with implant-supported restoration. Clinical evaluation revealed an asymptomatic, soft-consistency, red/purple tumor-like mass with a broad base, measuring 1.5-2 cm in diameter (Figure 1). 

**Figure 2:**
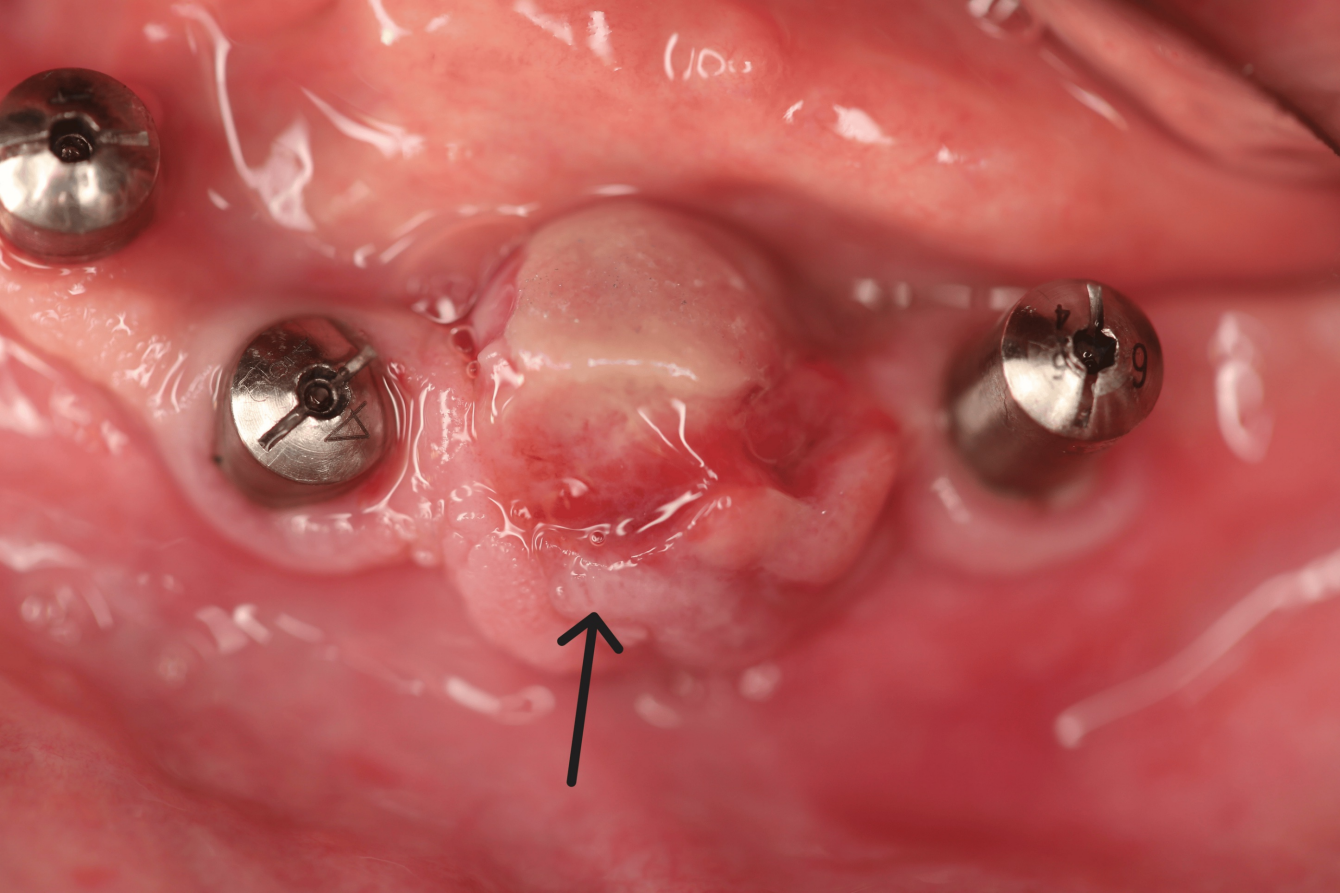
Clinical image of the lesion before the removal of the prosthetic restoration.

The lesion was located on both the buccal and lingual aspects of the alveolar ridge in the mandibular region in positions #34-#35 (Figure 2). 

**Figure 3:**
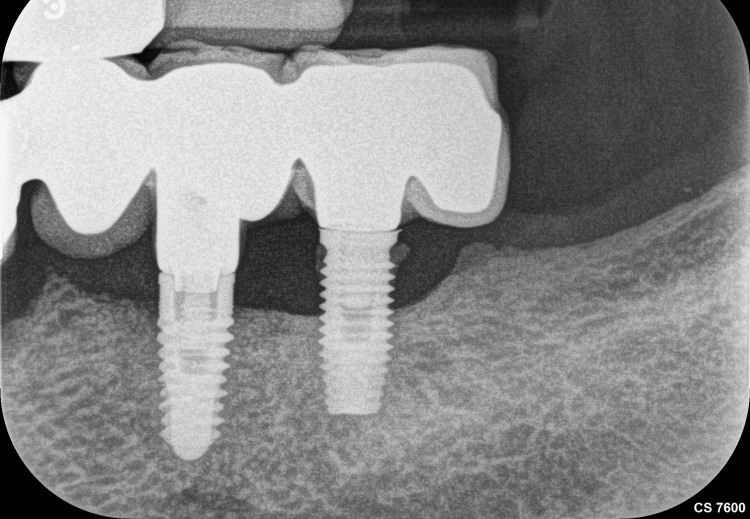
Radiographic evaluation revealed normal crestal bone height with mild bone loss involving two to three threads around each implant and the presence of calculus deposits on the distal surface of the implant. A periapical radiograph was acquired using a Carestream CS 7200 phosphor‑plate imaging system (Carestream Dental LLC, Atlanta, GA; size 2 plate). Exposure parameters: 70 kV, 7 mA, recommended exposure time 0.16 seconds for mandibular incisors/premolars as per manufacturer’s guidelines.

Radiographic evaluation with periapical radiographs revealed no significant findings (Figure 3).

**Figure 4:**
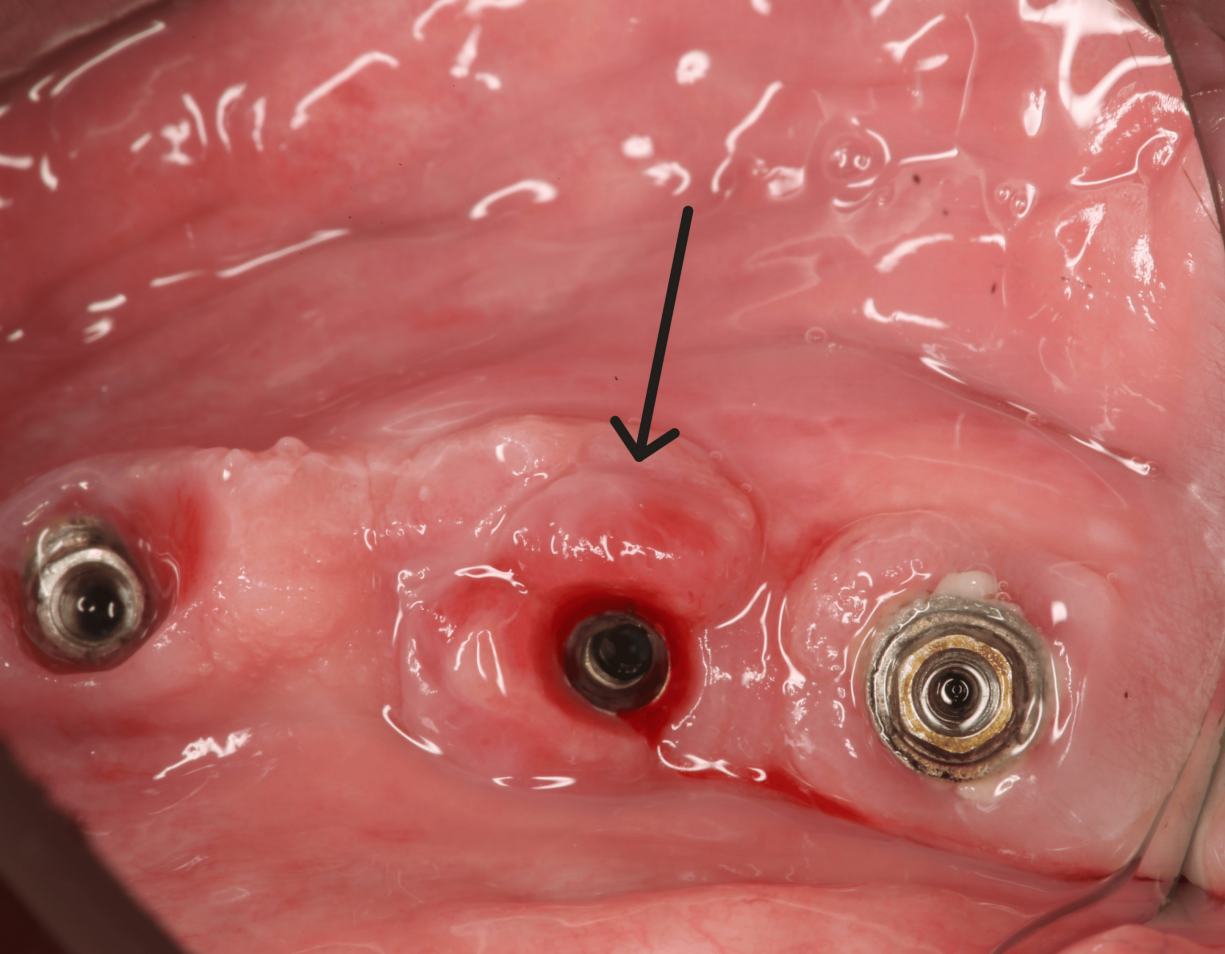
Surgical excision of the lesion followed by suturing of the surgical site.

The treatment involved a biopsy followed by surgical excision of the lesion (Figure 4) and subsequent bone curettage to minimize the risk of recurrence. Local anesthesia was administered using 1:100,000 lidocaine with adrenaline for vasoconstriction, without a nerve block. Due to the highly vascular nature of the granulation tissue, electrocautery was employed to control bleeding. Excision margins were carefully set in healthy tissue, at least 2 mm beyond the lesion, and primary closure was achieved with 5-0 absorbable polyglycolic acid (PGA) sutures. 

**Figure 5:**
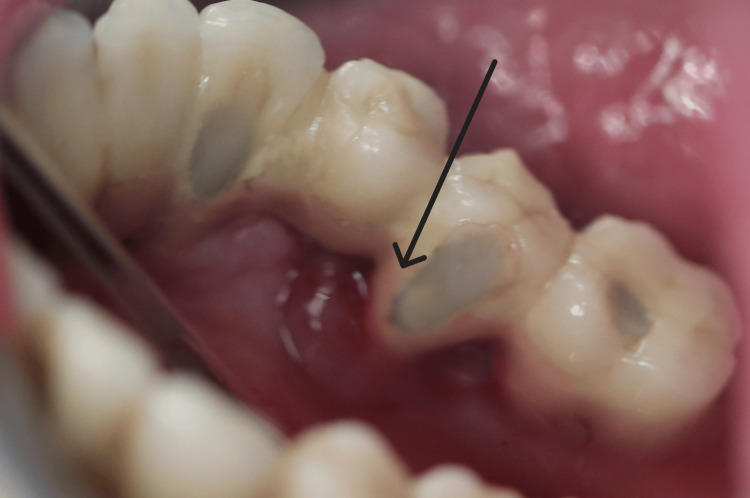
A clinical photograph obtained during the six-month follow-up after excision.

The excised tissue specimen was submitted for histopathological examination. Subsequently, the existing prosthetic restoration was modified and repositioned. According to the histopathological examination, a nodular formation consisting of aggregates of multinuclear giant cells within the chorion was observed. In this specific case, the overlying epithelium was not ulcerated. The microscopic evaluation of sections of the oral mucosa revealed a broadly ulcerated surface with underlying granulation tissue characterized by newly formed small blood vessels. No significant cellular atypia or mitotic activity is identified. In the deeper regions, the lesion demonstrates features consistent with giant cell changes, showing numerous multinucleated osteoclast-like giant cells within a stroma of intermediate cellularity composed of mononuclear histiocyte-like cells. In certain areas corresponding to the deeper portion of the specimen, trabecular bone fragments are also noted adjacent to the aforementioned stromal tissue. The differential diagnosis included giant cell granuloma, pyogenic granuloma, peripheral fibroma, and fibrous gingival hyperplasia. Based on the histopathological features, as well as clinical and radiographic examinations, the lesion was diagnosed as a PGCG. During the six-month follow-up examination, no signs of lesion recurrence were observed (Figure 5). 

The follow-up frequency of patients after biopsy and excision of PGCG in proximity to dental implants has not been standardized in clinical guidelines. However, evidence from the literature and large case series suggests that close and long-term monitoring is required due to the relatively high recurrence rate. At each follow-up visit, clinical evaluation is recommended to assess for signs of local recurrence, inflammation, and adequacy of keratinized mucosa, as well as radiographic examination to detect bone loss or peri-implant disease. At each follow-up visit, assessment of oral hygiene effectiveness should be performed. In cases where a decline is observed, the patient should be motivated and instructed accordingly, with the selection of the most appropriate oral hygiene interproximal brushes. This protocol should be performed every six months during the first year after lesion removal, since most recurrences are reported within the initial months [9-11]. If no recurrence is observed, follow-up intervals may then be extended to an annual basis for at least two to three years, as late recurrences beyond the first year have also been documented in Table 1 [9, 10, 12-14].

**Table 1:** Summary of the clinical timeline, treatment interventions, and follow-up for the reported implant-associated peripheral giant cell granuloma case.

Timepoint/Day	Clinical Stage/Observation	Therapeutic Intervention/Notes
Day 0	Initial visit: assessment	Clinical examination, medical and dental history, radiographic imaging
1.5 months	Lesion appearance/diagnosis	Biopsy and surgical excision of the lesion
Surgery day	Surgical procedure	Bone curettage, excision with ≥2 mm healthy tissue margins, hemostasis with electrocautery, closure with 5-0 absorbable PGA sutures
Three months	Postoperative follow-up	Clinical evaluation, assessment of healing
Six months	Follow-up	Clinical and radiographic evaluation for recurrence
12 months	Long-term follow-up	Assessment of healing and implant stability
24 months	Final review	Confirmation of absence of recurrence

## Discussion

PGCG is a benign fibrous connective tissue entity described as a tumor-like hyperplasia that exclusively appears on the gingiva and alveolar ridges. Histopathologically, PGCG is characterized by the presence of multinuclear giant cells and originates from periodontal membrane or periosteal cells. PGCG is considered a reactive lesion resulting from chronic traumatic or irritating factors, such as calculus accumulation, food impaction, or inadequate dental restorations.

The only available systematic review, conducted by Chrcanovic et al. (2019), reported a higher incidence of lesions in females compared to males (64.9%), in the mandible compared to the maxilla (67.6%), and in posterior regions compared to anterior regions (83.8%). Among the cases for which data were available, the average time interval between implant placement and lesion diagnosis was 41.0 ± 36.9 months (range: three to 134 months). Peri-implant bone loss was observed in 19 out of 23 cases (82.6%). In nine of 11 cases (81.8%) where implant surface information was reported, rough-surfaced implants had been used. The mean lesion size was 1.59 ± 1.16 cm (range: 0.4-6.0 cm; n = 32) [7].

Recurrence of PGCG was reported in nine out of 26 cases (34.6%), with a mean time to recurrence of 1.5 ± 1.7 months. Among lesions treated with surgical excision followed by curettage, recurrence occurred in five out of 16 cases (31.3%). In comparison, lesions managed with excision alone recurred in 4 out of 10 cases (40%). However, the difference in recurrence rates between the two treatment modalities was not statistically significant (P = 0.483). Notably, the recurrence rate of implant-associated PGCG (34.6%) is significantly higher than that reported for PGCG unrelated to dental implants, which stands at 9.5% [15]. 

Histopathological analysis revealed the presence of small black granular foreign material in 53.8% of the cases. Interestingly, these granules were not phagocytosed by multinucleated giant cells, as typically seen in foreign body granulomatous reactions, but were instead scattered throughout the stromal compartment without an associated inflammatory infiltrate, suggesting an inert behavior. Polarized light examination confirmed birefringence of the foreign particles in the majority of positive cases. It had previously been suggested that foreign material deposited in the stroma may play a role in the pathogenesis of implant-associated PGCG [16]. However, findings from the present and previous studies indicate that such particles, including metallic debris and titanium remnants, which have also been detected in other peri-implant lesions, are not unique to PGCG and are unlikely to play a significant pathogenetic role in this entity [16]. Furthermore, 80% of the lesions were associated with implants that had a rough surface [15], supporting the notion that implant surface characteristics may contribute to lesion development, although the presence of foreign material alone does not appear to be a determining factor.

It has been suggested that the true incidence of PGCG associated with dental implants (implant-related PGCG) may be underestimated. A possible contributing factor is that peri-implant soft tissue lesions are sometimes excised without being submitted for histopathological examination. Moreover, although a foreign body reaction exhibits distinct clinicopathological features compared to giant cell lesions of the jaws, the two entities may present with similar clinical appearances, leading to excision of the lesion without further investigation through biopsy. Since PGCG can be difficult to differentiate from other reactive lesions, such as pyogenic granuloma, histopathological evaluation remains essential. In pyogenic granuloma, vascular hyperplasia and absence of giant cells are observed, which distinguishes it from PGCG. The differential diagnosis from central giant cell granuloma (CGCG) is mainly based on location, as CGCG arises intraosseously, often demonstrating aggressive behavior and radiographic findings of intraosseous lesions, in contrast to PGCG, which is extraosseous [1]. Similarly, giant cell tumor of bone exhibits a distribution of giant cells within the stroma, is usually symptomatic, and carries a risk of malignant transformation and metastasis [17]. By contrast, PGCG is considered benign but may exhibit locally aggressive behavior [12]. In general, any oral soft tissue lesion that persists for more than 14 days should be subjected to biopsy in order to establish a definitive diagnosis. In addition, regular follow-up is essential to assess the clinical course of the lesion and to rule out recurrence or malignant transformation. For the surgical management of soft tissues, the technique of apical mattress suturing has been shown to increase the width of keratinized mucosa after implant placement, which contributes to the long-term health of peri-implant tissues and facilitates oral hygiene [18]. Monitoring the width of keratinized mucosa is essential, as inadequate width is associated with an increased risk of inflammation and difficulty in cleaning. Regular clinical assessment and timely intervention to maintain or increase the width of keratinized mucosa are necessary to prevent peri-implant diseases [18].

Regarding prosthetic factors, the choice of abutment type affects postoperative bone resorption. The use of temporary abutments before the final restoration is associated with greater bone loss compared to the immediate placement of the final abutment, highlighting the importance of the "one abutment-one time" strategy for maintaining bone levels [19]. For peri-implant postoperative care, the topical application of hyaluronic acid has been documented to significantly reduce pain after implant placement, with no reported adverse effects, and can be incorporated as an adjunctive therapy to improve patient comfort [20]. Overall, the integration of evidence-based surgical techniques, systematic monitoring of the mucosa, rational selection of prosthetic components, and individualized postoperative care are key elements for the optimal management of peri-implant tissues.

## Conclusions

Although implant-related PGCG remains a rare complication in the context of widespread dental implant use, its true incidence may be underreported, partly due to the occasional omission of histopathological analysis following excision of peri-implant soft tissue lesions. Given the clinical similarities between PGCG and other reactive lesions, such as pyogenic granulomas, histopathological evaluation remains essential for accurate diagnosis and appropriate management. These observations highlight the importance of clinician awareness in recognizing suspicious peri-implant lesions and in adopting surgical protocols that include complete excision with curettage and histological examination. Based on the literature, we recommend follow-up visits at three, six, and 12 months postoperatively to monitor for potential recurrence. Further studies with larger sample sizes are warranted to better understand the etiopathogenesis and optimal management strategies for implant-associated PGCG.
